# Cluster of differentiation 45 activation is crucial in interleukin-10-dependent tumor-associated dendritic cell differentiation

**DOI:** 10.3892/ol.2014.2161

**Published:** 2014-05-19

**Authors:** DA-EN CHENG, YING-MING TSAI, YA-LING HSU, MING-FENG HOU, EING-MEI TSAI, JAW-YUAN WANG, JUNG-YU KAN, PO-LIN KUO

**Affiliations:** 1 Graduate Institute of Medicine, College of Medicine, Kaohsiung Medical University, Kaohsiung 807, Taiwan, R.O.C.; 2 Division of Pulmonary and Critical Care Medicine, Kaohsiung Medical University Hospital, Kaohsiung 807, Taiwan, R.O.C.; 3 Institute of Clinical Medicine, College of Medicine, Kaohsiung Medical University, Kaohsiung 807, Taiwan, R.O.C.; 4 Department of Medical Research, Kaohsiung Medical University Hospital, Kaohsiung 807, Taiwan, R.O.C.

**Keywords:** cluster of differentiation 45, interleukin-10, monocytes, tumor-associated dendritic cells

## Abstract

Tumor-associated dendritic cells (TADCs) are important in tumor immune surveillance, and it has been reported that the secretion of interleukin (IL)-10 by cancer cells is a major factor involved in the induction of TADCs in the tumor microenvironment. In the present study, IL-10 was found to activate cluster of differentiation (CD)45 protein tyrosine phosphatase (PTPase), inducing a TADC-like phenomenon. The PTPase inhibitor, phenylarsine oxide, and a CD45 inhibitor reversed the IL-10-induced impaired differentiation of the DCs, and also reversed the induction of the TADCs by A549, MDA-MB-231 and SW480 conditioned media, which thus represents a novel therapy to reduce immune surveillance in the tumor microenvironment. The present study is the first to identify that CD45 is involved in IL-10-activated signaling in myeloid lineage cells.

## Introduction

Dendritic cells (DCs) are antigen-presenting cells with the unique ability to take up and process antigens in the peripheral blood and tissues (1). Subsequent migration to the draining lymph nodes then occurs, where the antigens are presented to resting lymphocytes. Antigen ingestion and processing is particularly efficient within immature DCs, however, for an efficacious T-cell response, maturation to fully activated DCs must occur; these cells express high levels of cell-surface major histocompatibility complex (MHC)-antigen complexes and costimulatory molecules. Tumor-associated dendritic cells (TADCs) are DCs that present in the tumor microenvironment with tolerogenic or suppressive functions, which exhibit lower expression levels of costimulatory and MHC molecules (2). TADCs have also been associated with a higher expression level of tolerogenic mediators, including indoleamine-2,3-dioxygenase, arginase and transforming growth factor (TGF)-β (3). In addition to inducing immune surveillance, TADCs have been reported to secrete factors that increase cancer progression (4–6).

Previous studies have shown that tolerogenic DCs (tDCs) may be induced by the presence of interleukin (IL)-10 in culture medium or in the tumor microenvironment (7–9). The binding of IL-10 and the IL-10 receptor has been reported to trigger several downstream pathways in mononuclear cells, including the Janus kinase (JAK)-1/signal transducer and activator of transcription-3 (STAT-3) (10,12), p38 mitogen-activated protein kinase, extracellular signal-regulated kinase and c-Jun N-terminal kinase signaling pathways (12). Among the signaling induced by IL-10, the JAK/STAT3 signaling pathway has been reported to be regulated by a group of protein tyrosine phosphatases (PTPases), termed suppressors of cytokine signaling (13,14).

Cluster of differentiation (CD)45 is a member of the PTPase family that is distributed in the plasma membrane of hematopoietic cells, with the exception of erythrocytes and platelets, and exhibits a crucial function in T-cell receptor-mediated signaling. Previous studies have shown that CD45 also regulates JAK (15–17) and Src (18,19) families. In addition, the negative regulation of cytokine receptor signaling by CD45 may explain the loss of CD45 activity observed in several cancer types, including leukemia. CD45 has been found to correlate with the proliferation of myeloma cells, and it may therefore present a potential target for the treatment of multiple myelomas (20). Despite its abundant expression, the function of CD45 in cells of the myeloid lineage is poorly understood. Fulcher *et al* (21) reported that CD45 may be a receptor for galectin-induced cell activation and migration via Syk and protein kinase C signaling in DCs. Previous studies regarding the function of CD45 in cancer have focused on myelomas (22,23), however, none of these studies have investigated the function of CD45 in tumor immune surveillance. The present study demonstrated that CD45 activation is important in tumor- and IL-10-induced tDCs.

## Materials and methods

### Cell lines, cell cultures and condition medium collection

**Figure 1:**
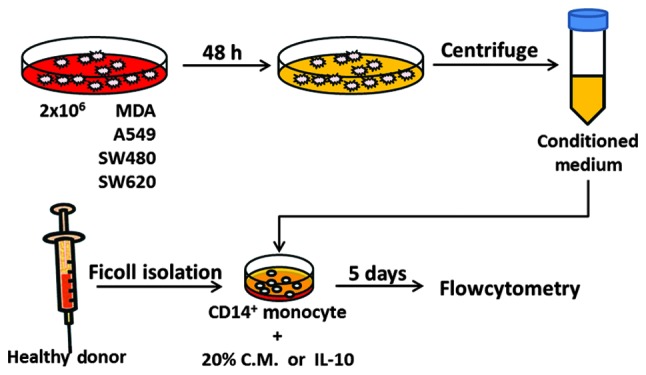
Diagram showing the conditioned medium transfer system. A total of 2×10^6^ cancer cells were plated in a 10-cm dish and incubated for 48 h. The supernatant was transferred to a 50-ml centrifuge tube and centrifuged at 1,000 × g for 15 min. CD14^+^ monocytes were isolated from healthy donor peripheral blood mononuclear cells and incubated with 20 ng/ml granulocyte-macrophage colony-stimulating factor and IL-4 alone or with an additional 20% cancer cell-conditioned medium or 10 ng/ml IL-10 for five days. IL, interleukin; CD14, cluster of differentiation 14.

Human breast adenocarcinoma MDA-MB-231 cells (HTB-26) and human lung adenocarcinoma A549 cancer cells (CCL-185) were obtained from the American Type Culture Collection (Manassas, VA, USA). The human colorectal adenocarcinoma SW620 cells (BCRC 60343) and SW480 (BCRC 60249) cells were purchased from the Bioresource Collection and Research Center (Hsinchu City, Taiwan). The human lung adenocarcinoma A549 cancer cells were cultured in minimum essential medium (Life Technologies, Inc., Grand Island, NY, USA) with 10% fetal bovine serum (FBS), non-essential amino acids and 0.1 mM sodium pyruvate (all Thermo Fisher Scientific, Waltham, MA, USA). The human breast adenocarcinoma MDA-MB-231 cells were cultured in F12K medium (Life Technologies, Inc.) with 10% FBS, non-essential amino acids and 0.1 mM sodium pyruvate. The SW620 and SW480 human colorectal adenocarcinoma cells were cultured in Leibovitz’s L-15 medium (Life Technologies, Inc.) supplemented with 10% FBS. The medium was changed once every 2–3 days and the cells were channeled once a distinct cell density had been reached. To obtain the conditioned medium, 2×10^6^ cells/well were seeded in a 100 mm dish and cultured for 24 h. The medium was then replaced and the supernatants were harvested following 48 h of incubation (Fig. 1).

### Reagents and antibodies

Recombinant human granulocyte-macrophage colony-stimulating factor (GM-CSF) and IL-4 were purchased from Millipore (Bedford, MA, USA). Recombinant human IL-10 was purchased from R&D Systems (Minneapolis, MN, USA). Phenylarsine oxide (PAO), CD45 PTPase inhibitor and PTPase inhibitor XVIII were purchased from Merck Millipore (Bedford, MA, USA). Fluorescein isothiocyanate-conjugated anti-CD16, phycoerythrin-conjugated anti-CD163, anti-CD11c, -CD80, -CD1a and -CD14, and APC-conjugated anti-CD14 monoclonal antibodies (mAbs) were purchased from BD Pharmingen (San Diego, CA, USA).

### Monocyte isolation and differentiation

Mononuclear cells were isolated from the blood of healthy donors using the Ficoll-Hypaque gradient (GE Healthcare, Little Chalfont, UK). Approval for this study was obtained from the Institutional Review Board of Kaohsiung Medical University Hospital (Kaohsiung, Taiwan), and informed consent was obtained from all patients in accordance with the Declaration of Helsinki. CD14^+^ monocytes were purified using CD14^+^ mAb-conjugated magnetic beads (MACS MicroBeads; Miltenyi Biotec, Bisley, UK) according to the manufacturer’s instructions. A control group of monocyte-derived dendritic cells was generated by culturing CD14^+^ monocytes in RPMI 1640 medium containing 10% FBS (Invitrogen Life Technologies, Carlsbad, CA, USA), 20 ng/ml GM-CSF and 20 ng/ml IL-4 (Millipore) for five days. In the IL-10, A549, SW480, SW620 and MDA groups, an additional 10 ng/ml IL-10 (R&D Systems) or 20% cancer cell-conditioned medium was added. The medium was replaced with fresh medium containing GM-CSF and IL-4 on day three. Following five days of incubation, the presence of CD14 and other surface markers was determined by fluorescence-activated cell sorting array flow cytometry using fluorochrome-conjugated mAbs (BD Pharmingen) (Fig. 1).

### Assessment of CD45 PTPase activity

CD14^+^ monocytes were incubated in RPMI containing GM-CSF and IL-4 with or without IL-10 for 30 min. The activity of CD45 PTPase was determined by a Human Active CD45 activity assay (R&D Systems) according to the manufacturer’s instructions. Briefly, 5×10^6^ CD14^+^ monocytes in 200 μl lysis buffer and 100 μl lysate were applied to the assay plate. Absorbance at 620 nm was measured using the Biotek Powerwave 340 ELISA reader (BioTek Instruments, Inc., Winooski, VT, USA).

### Statistical analysis

Data are presented as the mean ± standard deviation. Statistical analyses were performed by analysis of variance and two-sided t-tests using Excel 2010 (Microsoft, Tulsa, OK, USA). P<0.05 was considered to indicate a statistically significant difference between the means of the two test groups.

## Results

### IL-10 may induce TADC-like DCs

**Figure 2:**
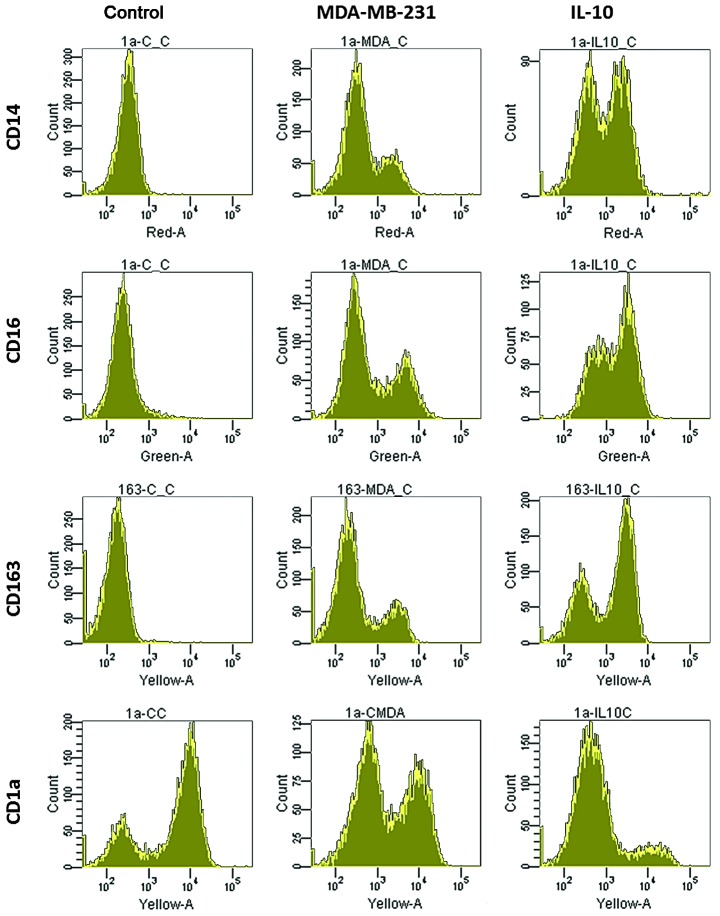
IL-10 induces tumor-associated dendritic cell-like surface marker expression. CD14^+^ monocytes were incubated with 20 ng/ml granulocyte-macrophage colony-stimulating factor and IL-4 alone or with an additional 20% MDA-MB-231-conditioned medium or 10 ng/ml IL-10 for five days. The cells were stained with antigen-presenting cell conjugated anti-CD14 mAb, fluorescein isothiocyanate-conjugated anti-CD16 mAb, PE-conjugated anti-CD11c mAb and PE-conjugated anti-CD1a mAb. The fluorescence intensity was determined by a fluorescence-activated cell sorting array flow cytometry system. All results are representative of at least three independent experiments. IL, interleukin; CD, cluster of differentiation; mAb, monoclonal antibody; PE, phycoerythrin.

The monocytes isolated from the healthy donors were treated with 20 ng/ml GM-CSF and IL-4 and incubated for five days for differentiation into resting DCs. In the MDA-MB-231 and IL-10 groups, an additional 20% of MDA-MB-231 breast cancer cell-conditioned medium and 10 ng/ml IL-10 was added. Following five days of incubation, the MCF and IL-10 groups exhibited a different expression pattern of surface markers when compared with the control group. In the control group, no CD14 expression was detected, however, 25% of the cells expressed CD14 in the MDA group. In addition, the CD14 expression was higher in the IL-10 group than in the MDA group. CD16 and CD163 expression showed the same pattern as CD14 in the three groups, in contrast to the expression of CD1a (Fig. 2). However, no significant differences were identified in the expression of CD80 and CD11c in the three groups (data not shown).

### CD45 PTPase is involved in IL-10-induced impaired DC differentiation

**Figure 3:**
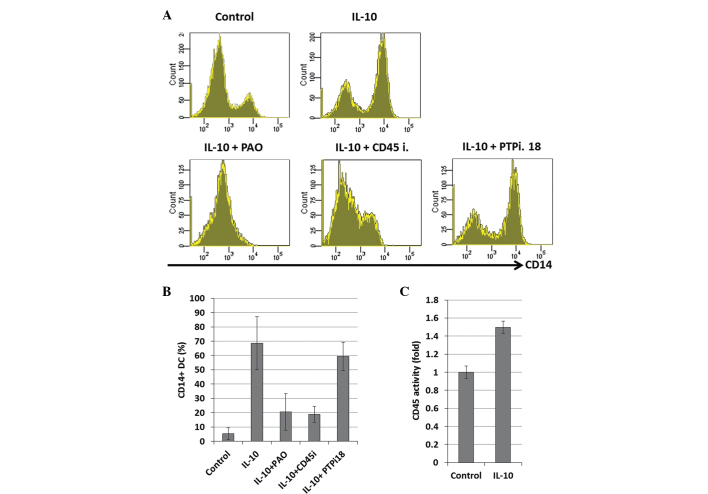
CD45 is involved in the IL-10 induction of tumor-associated dendritic cell-like DCs. (A and B) CD14^+^ monocytes were treated with 10 μM PAO, 100 nM CD45 PTPase inhibitor (CD45 i.) or 50 nM PTP inhibitor XVIII (PTPi. 18) for 1 h. The cells were later incubated with IL-10 (10 ng/ml) containing medium without or with (20 ng/ml) GM-CSF and IL-4 for five days. The surface expression of CD14 was assessed by phycoerythrin-conjugated anti-CD14 monoclonal antibody and a fluorescence-activated cell sorting array flow cytometry system. (C) CD14^+^ monocytes were incubated in RPMI containing GM-CSF and IL-4 with or without IL-10 for 30 min, and the activity of CD45 PTPase was determined using a CD45 Tyrosine Phosphatase assay kit. All results are representative of at least three independent experiments. Each value is presented as the mean ± standard deviation of three experiments. CD, cluster of differentiation; IL, interleukin; DC, dendritic cell; PTP, protein tyrosine phosphatase; GM-CSF, granulocyte-macrophage colony-stimulating factor; PAO, phenylarsine oxide.

To investigate whether the PTPases are involved in IL-10 signaling, three types of PTPase inhibitors were used in the present study. PAO is a general A membrane-permeable protein tyrosine phosphatase inhibitor, and PTP inhibitor XVIII inhibits the enzymatic activity of PTP1B, tyrosine-protein phosphatase non-receptor type 6, pathogenic *Yersinia* PTPase (YOP), T-cell protein tyrosine phosphatase, and yeast PTP1. PTP CD45 inhibitor (CD45i) is a selective and reversible inhibitor of CD45. Prior to the addition of IL-10 and GM-CSF/IL-4 to the culture medium, the CD14^+^ monocytes isolated from the healthy donors were incubated with the different inhibitors for 1 h. Following five days of incubation, the higher CD14 expression level mediated by IL-10 was reversed by the PAO and CD45 inhibitors, however, the CD14 expression of the DCs was not found to be downregulated by PTP inhibitor XVIII (Fig. 3A and B). The activation of CD45 was also confirmed by a CD45 tyrosine phosphatase assay kit (Enzo Life Sciences, Inc., Farmingdale, NY, USA), which showed that IL-10 activated CD45 phosphatase activity (Fig. 3C).

### PAO and CD45i reduces the number of tumor-mediated TADCs

**Figure 4:**
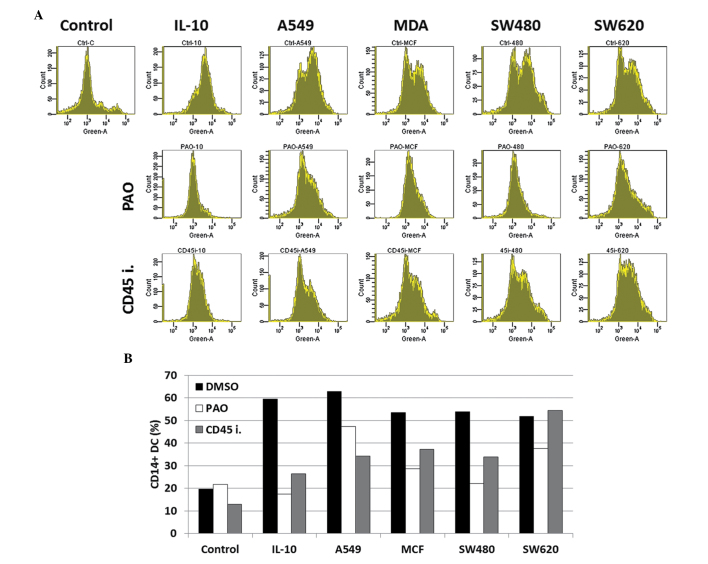
PAO and CD45 inhibitor reverse cancer cell-induced tumor-associated dendritic cells. (A and B) CD14^+^ monocytes were incubated in culture medium containing 20 ng/ml granulocyte-macrophage colony stimulating factor and IL-4 with or without different 20% cancer cell-conditioned media for 5 days. The surface expression of CD14 was assessed by phycoerythrin-conjugated anti-CD14 monoclonal antibody and a fluorescence-activated cell sorting array flow cytometry system. All results are representative of at least three independent experiments, one of which is shown. PAO, phenylarsine oxide; IL, interleukin; CD, cluster of differentiation; DMSO, dimethyl sulfoxide.

To investigate whether PAO and CD45i were able to block the differentiation of the TADCs, the conditioned medium of the varying cancer types, including that of the lung (A549), breast (MDA-MB-231) and colorectal cancer (SW480 and SW620) cells, was collected. The CD14^+^ monocytes isolated from the healthy donors were treated with PAO or CD45i prior to the addition of 20% cancer cell-conditioned medium. Following five days of incubation, the surface CD14 expression was determined, and it was found that PAO and CD45i reversed cancer cell-mediated TADC differentiation in the IL-10, A549, MDA and SW480 groups (Fig. 4A). However, only PAO, but not CD45i, reversed the induction of TADCs by SW620 (Fig. 4B).

## Discussion

DCs are antigen-presenting cells of hemopoietic origin with potent effects on primary T-cell differentiation and activation, and are thus of central relevance to antitumor immune responses and vaccine development (24,25). However, normal DC function is usually impaired in cancer patients (26,27). Previous studies have shown that immature or tDCs are induced by vascular endothelial growth factor and IL-10 (28–30), which is consistent with the results of the present study (Fig. 2). In the current study, cancer cell-conditioned medium and IL-10 induced higher expression levels of CD14, CD16 and CD163 with normal DC markers, including CD11c and CD209. This indicates that the TADCs or IL-10-induced DCs were similar to the circulating tDC-10 (31).

The IL-10 receptor (IL-10R) is a receptor tyrosine kinase, and the binding of IL-10 and IL-10R activates the JAK1 signaling pathway and its downstream factors, including STAT3, phosphoinositide 3-kinase (PI3K) and p38 (13,32,33). Notably, CD45 has been reported to not only regulate the JAK/STAT3 pathway (17), but also to activate p38 in B cells (34) and PI3K in monocytes (35). This indicates that CD45 may regulate the IL-10-mediated signaling pathway. The results of the present study also support the hypothesis that CD45 is crucial in the IL-10 signaling pathway. The general PTPase inhibitor, PAO, and CD45i reversed the IL-10-induced TADCs, however, PTP inhibitor XVIII was not found to block CD45 activity or the IL-10-mediated differentiation of the TADCs. These results also indicate that additional PTPases may not be involved in the IL-10 induction of TADCs. In addition, PAO and the CD45i were not found to impair normal DC differentiation (data not shown). PAO appeared to exhibit an increased efficacy in blocking the differentiation of TADCs, which may be due to the multiple targets of PAO, including internalization of ligand-receptor complexes (36), protein kinase C activity, phosphotyrosine phosphatase (37) and formyl peptide-stimulated and phorbol ester-stimulated phospholipase D (38). Therefore the present study indicates that PAO or a novel signaling pathway of IL-10 may represent a novel target.

Notably, PAO was found to completely block the differentiation of the TADCs induced by cancer cell-conditioned medium, however, CD45i was not found to block the induction of TADCs in SW620-conditioned medium. This suggests that SW620 may secrete factors other than IL-10 to induce TADCs. The comparison between SW620 and SW480 is a well-established model used to study the metastasis of colorectal cancer, whereby SW620 cells are isolated from the metastatic lymph nodes of the patients from whom SW480 cells are isolated. Furthermore, secretome studies have revealed that SW620 cells may secrete trefoil factor 3, growth/differentiation factor 15 (39), chemokine (C-X-C motif) ligand 8 (40), galectin-1 (41), TGFs and platelet-derived growth factor (42–44). Further studies to investigate the abilities of these factors in TADC induction are required, and the pathways also require clarification to prevent immune surveillance in distal metastatic organs. In conclusion, the present study is the first to demonstrate that CD45 PTPase activity is required for IL-10-mediated TADC differentiation, and that PAO and CD45 inhibitors block this process, which indicates that there is potential for the use of small molecules to modulate the immune system in the tumor microenvironment.

## References

[1] Steinman RM (1991). The dendritic cell system and its role in immunogenicity. Annu Rev Immunol.

[2] Nestle FO, Burg G, Fäh J, Wrone-Smith T, Nickoloff BJ (1997). Human sunlight-induced basal-cell-carcinoma-associated dendritic cells are deficient in T cell co-stimulatory molecules and are impaired as antigen-presenting cells. Am J Pathol.

[3] Watkins SK, Zhu Z, Riboldi E (2011). FOXO3 programs tumor-associated DCs to become tolerogenic in human and murine prostate cancer. J Clin Invest.

[4] Kuo CH, Chen KF, Chou SH (2013). Lung tumor-associated dendritic cell-derived resistin promoted cancer progression by increasing Wolf-Hirschhorn syndrome candidate 1/Twist pathway. Carcinogenesis.

[5] Kuo PL, Huang MS, Cheng DE, Hung JY, Yang CJ, Chou SH (2012). Lung cancer-derived galectin-1 enhances tumorigenic potentiation of tumor-associated dendritic cells by expressing heparin-binding EGF-like growth factor. J Biol Chem.

[6] Hsu YL, Huang MS, Cheng DE (2011). Lung tumor-associated dendritic cell-derived amphiregulin increased cancer progression. J Immunol.

[7] Han Y, Chen Z, Yang Y (2013). Human CD14^+^ CTLA-4^+^ regulatory dendritic cells suppress T cell response via cytotoxic T-lymphocyte antigen-4-dependent IL-10 and indoleamine-2,3-dioxygenase production in hepatocellular carcinoma. Hepatology.

[8] Lindenberg JJ, Oosterhoff D, Sombroek CC (2013). IL-10 conditioning of human skin affects the distribution of migratory dendritic cell subsets and functional T cell differentiation. PloS One.

[9] Kuo PL, Hung JY, Huang SK (2011). Lung cancer-derived galectin-1 mediates dendritic cell anergy through inhibitor of DNA binding 3/IL-10 signaling pathway. J Immunol.

[10] Lindenberg JJ, van de Ven R, Lougheed SM (2013). Functional characterization of a STAT3-dependent dendritic cell-derived CD14 cell population arising upon IL-10-driven maturation. Oncoimmunology.

[11] Donnelly RP, Dickensheets H, Finbloom DS (1999). The interleukin-10 signal transduction pathway and regulation of gene expression in mononuclear phagocytes. J Interferon Cytokine Res.

[12] Sato K, Nagayama H, Tadokoro K, Juji T, Takahashi TA (1999). Extracellular signal-regulated kinase, stress-activated protein kinase/c-Jun N-terminal kinase, and p38mapk are involved in IL-10-mediated selective repression of TNF-α-induced activation and maturation of human peripheral blood monocyte-derived dendritic cells. J Immunol.

[13] Staples KJ, Smallie T, Williams LM (2007). IL-10 induces IL-10 in primary human monocyte-derived macrophages via the transcription factor Stat3. J Immunol.

[14] Niemand C, Nimmesgern A, Haan S (2003). Activation of STAT3 by IL-6 and IL-10 in primary human macrophages is differentially modulated by suppressor of cytokine signaling 3. J Immunol.

[15] Porcu M, Kleppe M, Gianfelici V (2012). Mutation of the receptor tyrosine phosphatase PTPRC (CD45) in T-cell acute lymphoblastic leukemia. Blood.

[16] Xu D, Qu CK (2008). Protein tyrosine phosphatases in the JAK/STAT pathway. Front Biosci.

[17] Irie-Sasaki J, Sasaki T, Matsumoto W (2001). CD45 is a JAK phosphatase and negatively regulates cytokine receptor signalling. Nature.

[18] McFarland EC, Hurley TR, Pingel JT, Sefton BM, Shaw A, Thomas ML (1993). Correlation between Src family member regulation by the protein-tyrosine-phosphatase CD45 and transmembrane signaling through the T-cell receptor. Proc Natl Acad Sci USA.

[19] Hurley TR, Hyman R, Sefton BM (1993). Differential effects of expression of the CD45 tyrosine protein phosphatase on the tyrosine phosphorylation of the lck, fyn, and c-src tyrosine protein kinases. Mol Cell Biol.

[20] Bataille R, Robillard N, Pellat-Deceunynck C, Amiot M (2003). A cellular model for myeloma cell growth and maturation based on an intraclonal CD45 hierarchy. Immunol Rev.

[21] Fulcher JA, Chang MH, Wang S (2009). Galectin-1 co-clusters CD43/CD45 on dendritic cells and induces cell activation and migration through Syk and protein kinase C signaling. J Biol Chem.

[22] Descamps G, Pellat-Deceunynck C, Szpak Y, Bataille R, Robillard N, Amiot M (2004). The magnitude of Akt/phosphatidylinositol 3′-kinase proliferating signaling is related to CD45 expression in human myeloma cells. J Immunol.

[23] Ishikawa H, Mahmoud MS, Fujii R, Abroun S, Kawano MM (2000). Proliferation of immature myeloma cells by interleukin-6 is associated with CD45 expression in human multiple myeloma. Leuk Lymphoma.

[24] Timmerman JM, Levy R (1999). Dendritic cell vaccines for cancer immunotherapy. Ann Rev Med.

[25] Théry C, Amigorena S (2001). The cell biology of antigen presentation in dendritic cells. Curr Opin Immunol.

[26] Dunn GP, Bruce AT, Ikeda H, Old LJ, Schreiber RD (2002). Cancer immunoediting: from immunosurveillance to tumor escape. Nat Immunol.

[27] Almand B, Resser JR, Lindman B (2000). Clinical significance of defective dendritic cell differentiation in cancer. Clin Cancer Res.

[28] Gabrilovich DI, Chen HL, Girgis KR (1996). Production of vascular endothelial growth factor by human tumors inhibits the functional maturation of dendritic cells. Nat Med.

[29] Steinbrink K, Jonuleit H, Müller G, Schuler G, Knop J, Enk AH (1999). Interleukin-10-treated human dendritic cells induce a melanoma-antigen-specific anergy in CD8(+) T cells resulting in a failure to lyse tumor cells. Blood.

[30] Qin Z, Noffz G, Mohaupt M, Blankenstein T (1997). Interleukin-10 prevents dendritic cell accumulation and vaccination with granulocyte-macrophage colony-stimulating factor gene-modified tumor cells. J Immunol.

[31] Gregori S, Tomasoni D, Pacciani V (2010). Differentiation of type 1 T regulatory cells (Tr1) by tolerogenic DC-10 requires the IL-10-dependent ILT4/HLA-G pathway. Blood.

[32] Bhattacharyya S, Sen P, Wallet M, Long B, Baldwin AS, Tisch R (2004). Immunoregulation of dendritic cells by IL-10 is mediated through suppression of the PI3K/Akt pathway and of IkappaB kinase activity. Blood.

[33] Song GY, Chung CS, Schwacha MG, Jarrar D, Chaudry IH, Ayala A (1999). Splenic immune suppression in sepsis: A role for IL-10-induced changes in P38 MAPK signaling. J Surg Res.

[34] Arimura Y, Ogimoto M, Mitomo K (2001). CD45 is required for CD40-induced inhibition of DNA synthesis and regulation of c-Jun NH2-terminal kinase and p38 in BAL-17 B cells. J Biol Chem.

[35] Hayes AL, Smith C, Foxwell BM, Brennan FM (1999). CD45-induced tumor necrosis factor alpha production in monocytes is phosphatidylinositol 3-kinase-dependent and nuclear factor-kappaB-independent. J Biol Chem.

[36] Hoffman JF, Linderman JJ, Omann GM (1996). Receptor up-regulation, internalization, and interconverting receptor states Critical components of a quantitative description of N-formyl peptide-receptor dynamics in the neutrophil. J Biol Chem.

[37] Kutsumi H, Kawai K, Johnston RB, Rokutan K (1995). Evidence for participation of vicinal dithiols in the activation sequence of the respiratory burst of human neutrophils. Blood.

[38] Planat V, Tronchere H, Record M, Ribbes G, Chap H (1996). Involvement of vicinal dithiols in differential regulation of fMLP and phorbol ester-activated phospholipase D in stimulated human neutrophils. Biochem Biophys Res Commun.

[39] Dowling P, Clynes M (2011). Conditioned media from cell lines: a complementary model to clinical specimens for the discovery of disease-specific biomarkers. Proteomics.

[40] Bailey C, Negus R, Morris A (2007). Chemokine expression is associated with the accumulation of tumour associated macrophages (TAMs) and progression in human colorectal cancer. Clin Exp Metastasis.

[41] Ghosh D, Yu H, Tan XF (2011). Identification of key players for colorectal cancer metastasis by iTRAQ quantitative proteomics profiling of isogenic SW480 and SW620 cell lines. J Proteome Res.

[42] Ma C, Rong Y, Radiloff DR (2008). Extracellular matrix protein betaig-h3/TGFBI promotes metastasis of colon cancer by enhancing cell extravasation. Genes Dev.

[43] Coffey RJ, Shipley GD, Moses HL (1986). Production of transforming growth factors by human colon cancer lines. Cancer Res.

[44] Anzano MA, Rieman D, Prichett W, Bowen-Pope DF, Greig R (1989). Growth factor production by human colon carcinoma cell lines. Cancer Res.

